# Circulating Pancreatic Polypeptide Concentrations Predict Visceral and Liver Fat Content

**DOI:** 10.1210/jc.2014-3450

**Published:** 2014-12-09

**Authors:** Amir H. Sam, Michelle L. Sleeth, E. Louise Thomas, Nurhafzan A. Ismail, Norlida Mat Daud, Edward Chambers, Fariba Shojaee-Moradie, Margot Umpleby, Anthony P. Goldstone, Carel W. Le Roux, Paul Bech, Mark Busbridge, Rosemary Laurie, Daniel J. Cuthbertson, Adam Buckley, Mohammad A. Ghatei, Stephen R. Bloom, Gary S. Frost, Jimmy D. Bell, Kevin G. Murphy

**Affiliations:** Section of Investigative Medicine, Division of Diabetes, Endocrinology and Metabolism (A.S., C.W.L.R., P.B., R.L., A.B., M.A.G., S.R.B., K.G.M.), Imperial College London, United Kingdom; Nutrition and Dietetic Research Group, Section of Investigative Medicine, Division of Diabetes, Endocrinology and Metabolism (M.L.S., N.A.I., N.M.D., E.C., G.S.F.), Imperial College London, United Kingdom; Department of Life Sciences, Faculty of Science and Technology (E.L.S., J.D.B.), University of Westminster, London, United Kingdom; School of Chemical Sciences and Food Technology, Faculty of Science and Technology (N.M.D.), Universiti Kebangsaan Malaysia, Bangi, Selangor, Malaysia; Diabetes and Metabolic Medicine, Faculty of Health and Medical Sciences (F.S.M., M.U.), University of Surrey, Guildford, United Kingdom; Computational, Cognitive and Clinical Neuroimaging Laboratory, Division of Brain Sciences (A.P.G.), Imperial College London, United Kingdom; Diabetes Complications Research Centre (C.W.L.R.), Conway Institute, University College Dublin, Ireland; Department of Clinical Biochemistry (P.B., M.B.), Imperial College Healthcare NHS Trust, London, United Kingdom; and Department of Obesity and Endocrinology (D.J.C.), Institute of Ageing and Chronic Disease, University of Liverpool, United Kingdom

## Abstract

**Context and objective:**

No current biomarker can reliably predict visceral and liver fat content, both of which are risk factors for cardiovascular disease. Vagal tone has been suggested to influence regional fat deposition. Pancreatic polypeptide (PP) is secreted from the endocrine pancreas under vagal control. We investigated the utility of PP in predicting visceral and liver fat.

**Patients and Methods:**

Fasting plasma PP concentrations were measured in 104 overweight and obese subjects (46 men and 58 women). In the same subjects, total and regional adipose tissue, including total visceral adipose tissue (VAT) and total subcutaneous adipose tissue (TSAT), were measured using whole-body magnetic resonance imaging. Intrahepatocellular lipid content (IHCL) was quantified by proton magnetic resonance spectroscopy.

**Results:**

Fasting plasma PP concentrations positively and significantly correlated with both VAT (r = 0.57, *P* < .001) and IHCL (r = 0.51, *P* < .001), but not with TSAT (r = 0.02, *P* = .88). Fasting PP concentrations independently predicted VAT after controlling for age and sex. Fasting PP concentrations independently predicted IHCL after controlling for age, sex, body mass index (BMI), waist-to-hip ratio, homeostatic model assessment 2-insulin resistance, (HOMA2-IR) and serum concentrations of triglyceride (TG), total cholesterol (TC), and alanine aminotransferase (ALT). Fasting PP concentrations were associated with serum ALT, TG, TC, low- and high-density lipoprotein cholesterol, and blood pressure (*P* < .05). These associations were mediated by IHCL and/or VAT. Fasting PP and HOMA2-IR were independently significantly associated with hepatic steatosis (*P* < .01).

**Conclusions:**

Pancreatic polypeptide is a novel predictor of visceral and liver fat content, and thus a potential biomarker for cardiovascular risk stratification and targeted treatment of patients with ectopic fat deposition.

It is increasingly recognized that obesity is not a homogeneous condition and that cardiovascular risk can vary between individuals with a similar body mass index (BMI) (1). Variation in body fat distribution is an important determinant of cardiometabolic risk among patients with obesity. The intra-abdominal visceral deposition of fat is a major contributor to the development of insulin resistance, diabetes mellitus, hyperlipidemia, and hypertension (2). Visceral adipose tissue (VAT) and intrahepatocellular lipid content (IHCL) are independently and more strongly associated with an adverse metabolic risk profile than sc adipose tissue (3).

Regional body fat distribution and ectopic fat deposition can be identified using magnetic resonance imaging and proton magnetic resonance spectroscopy (4). However, such methods require significant technical and financial resources. There is therefore a need for more easily measured biomarkers that predict the extent of visceral and liver fat deposition, and which can thus be used to identify individuals at higher risk of metabolic or cardiovascular disease.

Pancreatic polypeptide (PP) is a member of the PP fold peptide family and is secreted postprandially from PP cells of the pancreatic islets of Langerhans. PP has been shown to inhibit food intake, gastric emptying, pancreatic exocrine secretion, and gallbladder contraction (5). PP secretion is thought to be primarily under vagal control (6). PP concentrations following an iv glucose injection have been reported to be weakly associated with intra-abdominal fat as measured by computed tomography in human subjects, although this association was not independent of age or sex (7). However, iv glucose has been reported to modulate circulating PP concentrations (8), and fasting PP concentrations may better reflect intra-abdominal vagal tone. Furthermore, intrahepatic fat has been suggested to be a better marker of obesity-associated metabolic complications than visceral fat (9). We hypothesized that variations in visceral parasympathetic activity would alter both VAT deposition and PP release, and thus that obese individuals with increased visceral and liver fat content could be identified by their elevated plasma PP concentrations.

## Materials and Methods

### Participants

Participants took part in studies at Imperial College London and University of Surrey that had all been approved by local Research and Ethics committees and were performed according to the principles of the Declaration of Helsinki between December 2007 and September 2012. Subjects were recruited through local advertising and from the obesity clinic. Exclusion criteria included diabetes mellitus, intercurrent/chronic medical or psychiatric illness, pregnancy, or alcohol or substance abuse. Written informed consent was obtained from all subjects. Anthropometric measurements (weight, height, waist, and hip circumference) were made, and BMI and waist-to-hip ratio (WHR) calculated.

### Biochemical measurements

Blood samples for PP measurement were collected, centrifuged at 4°C and plasma separated and stored at −20°C before being assayed in duplicate using an established in-house RIA in the Section of Investigative Medicine, Imperial College London (10) (Supplemental Data). To establish the potential variability of PP measurement in samples collected using different methods, we investigated the effect of the type of tube used for sample collection, time between blood collection and plasma/serum separation, and freeze-thaw cycles on plasma PP measurements. The type of tube used to collect blood samples (lithium heparin, lithium heparin tubes containing aprotinin [Trasylol], ethylenediaminetetraacetic acid, plain, and serum separation tubes), the time between blood collection and plasma and serum separation (up to 4 and 5 h, respectively) and freeze-thaw cycle number (up to four) had no significant effect on measured plasma PP concentrations (Supplemental Table 2 and Supplemental Figure 1).

Plasma insulin, glucose, cholesterol, triglycerides (TG), and alanine aminotransferase (ALT) concentrations were analyzed using an Abbott Architect ci8200 analyzer (Abbott Diagnostics) and Advia 1800 Chemistry System (Siemens Healthcare Diagnostics). Serum insulin was measured using an Abbott Architect ci8200 analyzer (Abbott Diagnostics) and a RIA kit (Millipore Corporation). Fasting insulin and glucose were used to calculate homeostatic model assessment 2-insulin resistance (HOMA2-IR) (11).

### Magnetic resonance imaging and spectroscopy of liver fat

Rapid T1-weighted magnetic resonance images were acquired using a 1.5T Phillips Achiva scanner (Phillips), as previously described (12). Total and regional adipose tissue volumes (sc and internal, both further separated into abdominal and nonabdominal compartments) were measured as previously defined (4, 12). Intra-abdominal adipose tissue is referred to as visceral adipose tissue. Intrahepatocellular lipid content (IHCL) was quantified by proton magnetic resonance spectroscopy as previously described (13).

### Statistical analysis

Analyses were performed using Prism version 5.1 software (GraphPad Software) and IBM SPSS Statistics version 22. Sample size calculation showed that 92 subjects were required for a power of 80%, significance level (α) of 0.05, nine independent variables, and a multiple regression coefficient (R) of 0.4. Normally distributed data are presented as mean ± SD and nonnormally distributed data as median (interquartile range). The student *t* test and Mann-Whitney *U* test were used to test differences between normally distributed and non-normally distributed data sets, respectively. Associations between plasma PP and BMI, total sc adipose tissue, VAT, IHCL, and fasting insulin concentrations were examined using Spearman's rank correlation. Data that were not normally distributed were log transformed when necessary. Multiple regression analysis was used to examine the association between fasting plasma PP and both VAT and IHCL, adjusting for a number of potential confounding variables. Logistic regression was used to examine the predictive ability of PP and HOMA2-IR in the diagnosis of hepatic steatosis. *P* < .05 was considered statistically significant.

## Results

Forty-six men and 58 women were studied. Demographic, anthropometric, and biochemical characteristics, and regional fat distributions of the men and women in the study population are described in Supplemental Table 1. Plasma PP concentrations correlated with VAT (r = 0.57, *P* < .001) and IHCL (r = 0.51, *P* < .001). The correlation between fasting PP and IHCL is shown in Supplemental Figure 1. There was a weak but significant correlation between PP and BMI (r = 0.24, *P* = .02), but not between PP and sc adipose tissue (r = 0.02, *P* = .88). There was a significant correlation between fasting PP and insulin concentrations (r = 0.34, *P* < .001) and between fasting insulin concentration and IHCL (r = 0.64, *P* < .001) and VAT (r = 0.55, *P* < .001), as expected. The correlation between fasting PP concentrations and VAT or IHCL remained significant after controlling for fasting plasma insulin concentrations (*P* < .001).

### PP and VAT

The association between fasting plasma PP concentrations and VAT was further analyzed, controlling for age, sex, and HOMA2-IR (Tables 1 and 2). The association between fasting plasma PP and visceral adipose tissue remained significant when age and sex were adjusted for in the analysis, but not after adjusting for HOMA2-IR (*P* = .07).

**Table 1.** Associations Between Fasting Plasma PP Concentrations and VAT, Adjusting for Age, Sex, and HOMA2-IR

Model	Adjustments	Coefficient	(95% CI)	*P* Value
1	None	0.35	(0.19–0.51)	<.001
2	Age	0.19	(0.04–0.34)	.02
3	Model 2 + sex	0.16	(0.03–0.29)	.02
4	Model 3 + HOMA2-IR	0.11	(−0.01–0.23)	.07

Abbreviation: CI, confidence interval.

The coefficients (and corresponding confidence intervals) indicate the change in VAT for a 10-pmol/L increase in fasting plasma PP concentrations.

**Table 2.** Associations between fasting plasma PP concentrations and IHCL, adjusting for age, sex, BMI, WHR, HOMA2-IR, and serum concentrations of TG, TC, and ALT

Model	Adjustments	Ratio	(95% CI)	*P* Value
**1**	None	1.28	(1.17–1.40)	<.001
**2**	Age, Sex	1.17	(1.08–1.27)	<.001
**3**	Model 2 + BMI, WHR, HOMA2-IR	1.11	(1.04–1.20)	.004
**4**	Model 3 + TG, total cholesterol	1.10	(1.03–1.18)	.005
**5**	Model 4 + ALT	1.12	(1.05–1.19)	.001

Abbreviation: CI, confidence interval.

Because IHCL was analyzed on the log scale, the effect sizes are reported in the form of ratios. The ratios (and corresponding confidence intervals) are reported for a 10-pmol/L increase in fasting PP concentration.

### PP and IHCL

Fasting plasma PP concentrations remained an independent predictor of IHCL when age, sex, BMI, WHR, HOMA2-IR, and serum concentrations of TG, TC, and ALT were controlled for (Table 2). Because IHCL was analyzed on the log scale, the size of the effect is reported as a ratio. Without any adjustments, a 10-pmol/L increase in PP was associated with a 28% increase in IHCL. After adjustments for all other variables, a 10-pmol/L increase in PP was associated with a 12% increase in IHCL (Table 2).

Despite having the same BMI (33.0 vs 32.9, *P* = .71), obese individuals with hepatic steatosis (n = 35, defined as an IHCL > 5.5%) (13, 14) had a significantly higher median fasting plasma PP than obese individuals without hepatic steatosis (n = 29, 34.84 vs 17.66 pmol/L, *P* = .0002).

### PP and cardiometabolic risk factors

Fasting plasma PP concentrations correlated with serum ALT, TG, TC, low-density lipoprotein cholesterol, high-density lipoprotein cholesterol, systolic blood pressure (BP) and diastolic BP when no adjustments were made, but not after adjusting for either or both IHCL or visceral fat (Supplemental Table 3).

### PP and HOMA2-IR: Independent predictors of hepatic steatosis

Table 3 shows the odds ratios (and corresponding confidence intervals) quantifying the association between each variable and the odds of hepatic steatosis. The area under the receiver operating characteristic (ROC) curve (AUC) for each model is reported in Table 3. Both PP and HOMA2-IR were independently significantly associated with hepatic steatosis. The area under the ROC curve (89%) was significantly higher for the combination of PP and HOMA2-IR than for either PP or HOMA2-IR alone.

**Table 3.** ORs (and Corresponding CIs) Quantifying the Association Between Fasting Plasma PP and HOMA2-IR and Hepatic Steatosis

Model	Variable	OR	(95% CI)	*P* Value	AUC	(95% CI)
1	PP	2.03	(1.47–2.81)	<.001	0.80	(0.71–0.88)
2	HOMA2-IR	6.74	(3.05–14.90)	<.001	0.83	(0.76–0.91)
3	PP	1.93	(1.33–2.80)	.001	0.89	(0.82–0.95)
	HOMA2-IR	6.99	(2.73–17.84)	<.001		

Abbreviation: CI, confidence interval; OR, odds ratio.

The ORs give the relative change in the odds of hepatic steatosis for a one-unit increase in HOMA2-IR and 10-unit increase in fasting PP. The area under the ROC curve (AUC) and corresponding CIs for each model are shown in the last column.

## Discussion

We investigated the relationship between fasting plasma PP concentrations, and regional fat distribution and liver fat content. Fasting plasma PP concentrations were significantly associated with visceral but not sc adipose tissue. Visceral abdominal adiposity is strongly related to cardiometabolic risk factors and the prevalence of cardiovascular disease (15).

In our study, the correlations between fasting plasma PP concentrations and visceral/liver fat were more significant than that between fasting plasma PP concentrations and BMI. Obese patients with hepatic steatosis had significantly higher fasting plasma PP concentrations. Our data suggest that PP is a marker of visceral/liver fat rather than of BMI per se.

Fasting PP concentrations are a predictor of liver fat. Ectopic fat in the liver may be more important than visceral fat in the determination of metabolically healthy individuals (16). Fatty liver is an independent predictor of type 2 diabetes (17). There is currently no single biomarker that can reliably detect liver fat, which is an independent risk factor for cardiovascular disease (18). A liver fat score incorporating information regarding waist circumference, serum TG, serum high-density lipoprotein cholesterol, BP, fasting plasma glucose, type 2 diabetes, fasting serum insulin, and liver transaminases has been reported to predict nonalcoholic fatty liver disease and liver fat content (19). Although we did not have data for all of the parameters required for calculation of this liver fat score from our study participants, and hence cannot compare its utility for predicting liver fat with that of fasting plasma PP concentration, it would be interesting to directly compare these methods in future studies. Circulating PP measurement was not significantly influenced by a range of different collection methods, suggesting the collection of samples suitable for PP measurement could be performed in a routine clinical setting. Pancreatic polypeptide concentrations were associated with a number of cardiometabolic risk factors, including low-density lipoprotein cholesterol, TG, and BP. These associations were mediated by visceral and/or liver fat. Unsurprisingly, HOMA2-IR, a surrogate of insulin resistance, was a predictor of hepatic steatosis. Interestingly, however, fasting PP was an independent predictor of liver fat.

The increased PP levels associated with increased VAT and IHCL may reflect increased abdominal parasympathetic outflow (20). It is also possible that plasma PP levels reflect basal insulin secretion, and that insulin drives adipogenesis in specific depots. However, the correlation between fasting PP concentrations and VAT or IHCL remained significant after controlling for fasting plasma insulin concentrations.

In conclusion, measurement of fasting plasma PP concentrations may be useful in the prediction of visceral and IHCL content. Additional work is required to determine whether fasting plasma PP can predict cardiovascular disease and help distinguish metabolically benign and healthy obesity from metabolically abnormal normal weight and obese subjects. Future studies could also investigate whether fasting PP concentrations can predict response to bariatric surgery.
